# Risk factors associated with preterm birth after IVF/ICSI

**DOI:** 10.1038/s41598-022-12149-w

**Published:** 2022-05-13

**Authors:** Jian Li, Jinhua Shen, Xiaoli Zhang, Yangqin Peng, Qin Zhang, Liang Hu, Christoph Reichetzeder, Suimin Zeng, Jing Li, Mei Tian, Fei Gong, Ge Lin, Berthold Hocher

**Affiliations:** 1 Key Laboratory of Study and Discovery of Small Targeted Molecules of Hunan Province, School of Medicine, Hunan Normal University, Changsha, China; 2 First People’s Hospital of Changde, Hunan, China; 3 Fifth Department of Medicine (Nephrology/Endocrinology/Rheumatology), University Medical Centre Mannheim, University of Heidelberg, Mannheim, Germany; 4 Institute of Pharmacy, Freie Universität Berlin, Berlin, Germany; 5 Reproductive and Genetic Hospital of CITIC-Xiangya, Changsha, China; 6 Peking University Shougang Hospital, Beijing, China; 7 Institute of Reproductive and Stem Cell Engineering, School of Basic Medical Science, Central South University, No. 88 Xiangya Road, Changsha, 410008 China; 8 Key Laboratory of Stem Cells and Reproductive Engineering, Ministry of Health, Changsha, China; 9 National Engineering and Research Center of Human Stem Cell, Changsha, China; 10 Institute for Nutritional Science, University of Potsdam, Nuthetal, Germany

**Keywords:** Physiology, Health care, Risk factors

## Abstract

In vitro fertilization/intracytoplasmic sperm injection (IVF/ICSI) is associated with an increased risk of preterm (33rd–37th gestational week) and early preterm birth (20th–32nd gestational week). The underlying general and procedure related risk factors are not well understood so far. 4328 infertile women undergoing IVF/ICSI were entered into this study. The study population was divided into three groups: (a) early preterm birth group (n = 66), (b) preterm birth group (n = 675) and (c) full-term birth group (n = 3653). Odds for preterm birth were calculated by stepwise multivariate logistic regression analysis. We identified seven independent risk factors for preterm birth and four independent risk factors for early preterm birth. Older (> 39) or younger (< 25) maternal age (OR: 1.504, 95% CI 1.108–2.042, P = 0.009; OR: 2.125, 95% CI 1.049–4.304, P = 0.036, respectively), multiple pregnancy (OR: 9.780, 95% CI 8.014–11.935, P < 0.001; OR: 8.588, 95% CI 4.866–15.157, P < 0.001, respectively), placenta previa (OR: 14.954, 95% CI 8.053–27.767, P < 0.001; OR: 16.479, 95% CI 4.381–61.976, P < 0.001, respectively), and embryo reduction (OR: 3.547, 95% CI 1.736–7.249, P = 0.001; OR: 7.145, 95% CI 1.990–25.663, P = 0.003, respectively) were associated with preterm birth and early preterm birth, whereas gestational hypertension (OR: 2.494, 95% CI 1.770–3.514, P < 0.001), elevated triglycerides (OR: 1.120, 95% CI 1.011–1.240, P = 0.030) and shorter activated partial thromboplastin time (OR: 0.967, 95% CI 0.949–0.985, P < 0.001) were associated only with preterm birth. In conclusion, preterm and early preterm birth risk factors in patients undergoing assisted IVF/ICSI are in general similar to those in natural pregnancy. The lack of some associations in the early preterm group was most likely due to the lower number of early preterm birth cases. Only embryo reduction represents an IVF/ICSI specific risk factor.

## Introduction

There are more than 15 million preterm infants every year among the world, accounting for 10% of the total newborn infants, of which the incidence of preterm delivery in China ranks the second. Preterm delivery accounts for more than 75% of perinatal morbidity and mortality worldwide^1^. Furthermore, those infants who do survive have higher rates of long-term morbidities, including cardiovascular diseases^2^ as well as neurologic and developmental disabilities, compared to infants born full term. Known maternal risk factors for preterm birth in the general population include having a previous premature birth, twin pregnancy, an interval of less than six months between pregnancies, history of multiple miscarriages or abortions, smoking cigarettes or using illicit drugs, cardio-metabolic diseases such as hypertension or diabetes, and infections, particularly of the amniotic fluid and lower genital tract^3–7^. Conceiving through in vitro fertilization represents another risk factor in a subgroup of women undergoing assisted reproduction technologies (ART). The risk factors increasing the likelihood for preterm birth in this particular population are, however, as of today, not well understood. Numerous studies^8^ analyzed maternal and offspring outcomes after ART, whereas larger sized studies focusing specifically on the risk factors for preterm birth and especially early preterm birth especially associated to poor offspring outcome are lacking. However, this is a clinically important topic, since the understanding of ART related risk factors for preterm birth might identify changeable factors offering potential treatment options to improve offspring short term and long-term outcome in women undergoing ART. The aim of the current study was to identify causes or medical reasons for preterm and early preterm birth in a large cohort of women who underwent ART.

## Methods

### Ethics, inclusion and exclusion criteria, data collection

The study was approved by the ethics committee of Reproductive & Genetic Hospital of Citic-Xiangya, Changsha, China (approval document number: LL-SC-2019-003). A total of 4349 infertile women who had undergone IVF/ICSI treatment and obtained live birth in the Reproductive & Genetic Hospital of Citic-Xiangya from January 1st, 2016 to December 30th, 2017 were enrolled.

Inclusion criteria were as follows:

- Women treated with super-ovulation protocols exactly as described previously ^9^
- Fresh embryo transfer recipients, who received IVF/ICSI treatment
- Giving live birth after ART

Exclusion criteria were as follows:

- Using donor sperms or donor eggs for ART
- Complete clinical data were not available
- Post-term birth (> 42nd week of gestation) cases were excluded
- Infertile couples with known female or male genetic causes of infertility

Gestational age was calculated by adding 2 weeks (14 days) to the number of days since fertilization^10^. Note: Gestational age was determined as the 17th day of gestation when a 6–8 cell embryo was transferred into the uterus and as the 19th day of gestation when a blastocyst was transferred.

Full-term birth was defined as a live birth with a gestational age between 37 but not over 42 weeks (37 weeks ≤ gestational age < 42 weeks). Preterm birth was defined as a live birth with a gestational age of at least 20 but not over 37 weeks (20 weeks ≤ gestational age < 37 weeks)^11^. Early preterm birth was defined as a live birth with a gestational age between 20 but not over 32 weeks (20 weeks ≤ gestational age < 32 weeks)^12^.

All patient’s data (clinical data as well as laboratory data) used in our study were extracted from the routine electronic patient records used in our hospital.

### Clinic data collection

Informed consent was obtained from all subjects and/or their legal guardian(s). A structured medical history was taken. The following risk factors for preterm birth were examined in this study:

Maternal risk factors: (1) basic parameters before super-ovulation: nationality, education, age, body height, body weight, infertility duration, types of infertility, causes of infertility (maternal causes, paternal causes, maternal and paternal causes, unknown causes), blood pressure readings. (2) Pregnancy history: parity, artificial abortion, drug abortion, spontaneous abortion, ectopic pregnancy, number of deliveries, vaginal delivery, cesarean section (3) blood test result before super-ovulation: liver and kidney function, lipid items, blood coagulation function. (4) Pregnancy related factors: multiple pregnancy, embryo reduction, gestational diabetes, gestational hypertension, placenta previa.

Relevant risk factors during IVF/ICSI procedure: cycle count, fertilization way, embryo transfer type (blastocyst or cleavage stage embryo transfer), ovulation induction scheme, source of sperm, transferred embryo count, dosage of gonadotropin, ovulation inducing days.

Offspring data: gestational age at delivery, gender, birth weight.

Basic parameters about the mother, pregnancy history, gynecological complications, and relevant risk factors during IVF/ICSI procedure came from the case report in the hospital. Furthermore, blood test results from maternal blood taken before the beginning of superovulation was extracted from the case report. Pregnancy related factors and offspring data were followed up strictly by a special nurse.

### Patient and public involvement

This study is a retrospective study. Data were obtained through the electronical medical record system of the hospital. Patients were not directly involved in this study. The patients were unaware of the results of the study.

### Statistical analysis

Continuous variables are represented as mean ± SD for normally distributed variables and student’s unpaired t-test was used for comparison of variables between two groups. Continuous variables are represented as median and quartiles M (Q_1 _− Q_3_) for non-normally distributed variables and Mann–Whitney nonparametric test was used for comparison of variables between groups. Categorical variables are described as frequency and percentages. Pearson’s chi-square test was used for testing qualitative data and Fisher's exact test was used when the expected frequencies were < 5%.

Multivariate logistic regression analyses with step forward selection using the likelihood method were applied to examine the association between the patient’s characteristics and the risk of preterm brith. Analyzed variables with *P* < 0.05 in the univariate analysis were entered into the multivariate analysis. No other factors were considered as confounders. Results are represented as ORs with corresponding 95% CIs and *P* values.

Statistical package for social sciences (SPSS version 22.0, Chicago, IL, USA) was used to perform all data analyses and a two-sided *P* value < 0.05 was considered to be statistically significant.

All methods were carried out in accordance with relevant guidelines and regulations of the People Republic of China.

### Ethics approval

This study was approved by the ethics committee of the Reproductive and Genetic Hospital of CITIC-Xiangya, Changsha, China (approval number: ll-sc-2019-003). The data of this study is only used for this study, and the data of patients are strictly confidential. This study will not cause any harm to the patients' body and mind.

## Results

### Description of the cohort

**Figure 1:**
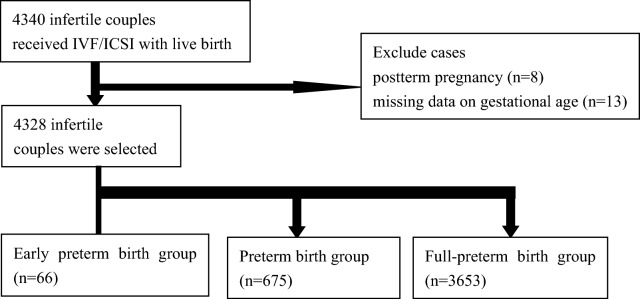
Study flow chart. Early preterm birth group: 20 weeks ≤ gestational age < 32 weeks; preterm birth group: 20 weeks ≤ gestational age < 37 weeks; full-term birth group: 37 weeks ≤ gestational age < 42 weeks.

From the primary dataset of 4349 treated women, we excluded 13 cases in which the gestational age was unclear and 8 cases that were post-term pregnancies. Thus finally 4328 cases were included into the study, 3653 of them were full-term deliveries. The prevalence of preterm birth and early preterm birth was 15.5% and 1.8% respectively (Fig. 1). The median age of the participating women was 30 (27, 33) years old. The median BMI of the women was 21.94 (20.08, 23.76) kg/m^2^. The study cohort consisted mostly of Han Chinese women (87.8%). Caused for infertility in the entire study populations were distributed as follows: primary infertility was present in 42.9% and secondary infertility in 57.1%. The duration of infertility was 4 (2, 6) years. Median birthweight was 3100 (2700, 3500) g, Median gestational age was 38.4 (37.3, 39.4) weeks. For more details see Tables 1 and 2.

**Table 1:** Basic parameters before super-ovulation were compared between preterm birth group and full-term birth group.

Variables	Preterm birth (n = 675)	Early-preterm birth (n = 66)	Full-term birth (n = 3653)	*P*^a^	*P*^b^
**Maternal age (year)**				0.026	0.021
25–39	599 (88.7%)	55 (83.3%)	3339 (91.4%)		
≥ 40 or 20–24	76 (11.3%)	11 (16.7%)	314 (8.6%)		
**Maternal BMI (kg/m**^**2**^**)**				0.342	0.506
< 18.5	57 (8.4%)	7 (10.6%)	301 (8.2%)		
18.5–23.99	455 (67.4%)	41 (62.1%)	2523 (69.1%)		
24–27.99	139 (20.6%)	15 (22.7%)	741 (20.3%)		
≥ 28	24 (3.6%)	3 (4.5%)	87 (2.4%)		
**Maternal nationality**				0.055	0.642
Han	593 (90.4%)	59 (90.8%)	3115 (87.3%)		
Non-Han	63 (9.6%)	6 (9.2%)	455 (12.7%)		
**Maternal education level**				0.342	0.473
Junior middle school and below	259 (39.7%)	34 (53.1%)	1439 (40.4%)		
Senior high school or technical secondary school	192 (29.4%)	15 (23.4%)	999 (28.0%)		
Junior college and above	201 (30.8%)	15 (23.4%)	1125 (31.6%)		
**Infertility period (year)**				0.694	0.156
1–4	426 (63.1%)	37 (56.1%)	2287 (62.6%)		
5–9	201 (29.8%)	21 (31.8%)	1132 (31.0%)		
≥ 10	48 (7.1%)	8 (12.1%)	234 (6.4%)		
**Infertility type**				0.269	0.642
Primary infertility	303(44.9%)	30 (45.5%)	1556 (42.6%)		
Secondary infertility	372 (55.1%)	36 (54.5%)	2097 (57.4%)		
Maternal diastolic blood pressure	70 (76–80)	70 (77–81)	70 (75–80)	0.125	0.086
Maternal systolic blood pressure	109 (113–120)	107.5 (113–122)	109 (114–120)	0.949	0.896

*BMI* body mass index.

^a^Preterm birth group vs.full-term birth.

^b^Early-preterm birth group vs.full-term birth.

**Table 2:** Baseline maternal blood test results were compared between preterm birth group and full-term birth group.

Variable	Preterm birth (n = 631)	Early-preterm birth (n = 66)	Full-term birth (n = 3374)	*P*^a^	*P*^b^
Alanine transaminase (U/L)	11.8 (15.4–22.2)	12.275 (15.45–19.95)	11.4 (15.0–20.8)	0.077	0.532
Glutamic oxalacetic transaminase (U/L)	15.9 (18.5–22.1)	16.725 (19.4–23.125)	15.8 (18.2–21.5)	0.089	0.056
Total bile acid (µmol/L)	0.7 (1.3–2.7)	0.9 (1.3–2.2)	0.7 (1.3–2.6)	0.472	0.580
Serum total protein (mg/L)	74.8 (77.7–80.3)	74.6 (77.25–80.375)	74.6 (77.2–79.9)	0.082	0.996
Serum albumin (g/L)	46.3 (48.3–50.1)	46.075 (48.5–49.95)	46.3 (48.0–49.7)	0.059	0.526
Serum globulin (g/L)	26.9 (29.2–31.4)	27.15 (29.05–31.225)	27.0 (29.1–31.3)	0.366	0.835
Total bilirubin (µmol/L)	7.378 (9.5–12.2)	7.775 (9.85–12.725)	7.2 (9.3–12.4)	0.734	0.316
Direct bilirubin (µmol/L)	3.0 (3.7–4.6)	3.075 (3.8–4.925)	3.0 (3.8–4.7)	0.493	0.579
Blood urea nitrogen (mmol/L)	3.4 (4.0–4.7)	3.30 (3.95–4.70)	3.3 (4.0–4.7)	0.780	0.772
Creatinine (µmol/L)	54.0 (59.0–65.0)	52.0 (58.5–66.25)	54 (60–66)	0.403	0.644
Uric acid (µmol/L)	228. (266–309.5)	232.0 (266.5–297.0)	227 (263–302)	0.256	0.732
Cholesterol (mmol/L)	4.00 (4.48–4.94)	3.745 (4.27–5.01)	3.93 (4.39–4.90)	0.022	0.572
Triglyceride (mmol/L)	0.71 (0.98–1.39)	0.7325 (1.04–1.3975)	0.69 (0.91–1.30)	0.009	0.301
High density lipoprotein (mmol/L)	1.25 (1.48–1.79)	1.23 (1.42–1.63)	1.28 (1.50–1.76)	0.628	0.134
Low density lipoprotein (mmol/L)	2.32 (2.74–3.29)	2.155 (2.65–3.6275)	2.26 (2.68–3.17)	0.022	0.957
Apolipoprotein-A1 (g/L)	1.4 (1.5–1.7)	1.3 (1.5–1.6)	1.4 (1.5–1.7)	0.915	0.027
Apolipoprotein-B (g/L)	0.70 (0.8–1.0)	0.6 (0.8–1.0)	0.7 (0.8–0.9)	0.010	0.989
Prothrombin time (s)	9.9 (10.40–10.91)	9.9 (10.4–10.9)	10 (10.4–10.9)	0.945	0.746
International normalized ratio	0.92 (0.96–1.00)	0.92 (0.955–1.0)	0.92 (0.96–1.00)	0.115	0.507
Activated partial thromboplastin time (s)	31.6 (34.5–37.58)	31.55 (35.0–37.6)	32.4 (35.2–38.1)	0.001	0.539
Fibrinogen (g/L)	2.33 (2.64–2.975)	2.4 (2.69–3.005)	2.33 (2.64–2.94)	0.624	0.660
Thrombin time (s)	12.6 (13.3–14.1)	12.4 (13.1–13.65)	12.6 (13.3–14.2)	0.666	0.041

^a^Preterm birth group vs. full-term birth.

^b^Early-preterm birth group vs. full-term birth group.

Univariate analysis showed that 15 parameters were significantly different between the full-term birth group and the preterm birth group (*P* < 0.05), including 6 maternal parameters (age, apolipoprotein B, total cholesterol, triglycerides, low density lipoprotein and activated partial thromboplastin time), 5 pregnancy related factors (multiple pregnancy, embryo reduction, placenta previa, gestational diabetes and gestational hypertension), 2 factors related to the IVF/ICSI procedure (embryo transfer type and number of embryos transferred) and 2 offspring related factor (infant sex and intrauterine growth retardation) (Tables 1, 2, 3).

**Table 3:** Pregnancy factors after embryo implantation was compared between preterm birth group and full-term birth group.

Variables	Preterm birth (n = 675)	Early-preterm birth (n = 66)	Full-term birth (n = 3653)	*P*^a^	*P*^b^
**Treatment cycle**				0.488	0.033
1	500 (74.1%)	45 (68.2%)	2763 (75.6%)		
2	144 (21.3%)	21 (31.8%)	754 (20.6%)		
≥ 3	31 (4.6%)	0 (0.0%)	136 (3.7%)		
**Fertilization method**				0.169	0.554
IVF	465 (68.9%)	45 (68.2%)	2612 (71.5%)		
ICSI	210 (31.1%)	21 (31.8%)	1041 (28.5%)		
**Embryo transfer type (%)**				< 0.001	0.027
Blastocyst transfer	58 (8.6%)	3 (4.5%)	512 (14%)		
Cleavage stage embryo transfer	617 (91.4%)	63 (95.5%)	3141 (86%)		
**Ovulation stimulation protocol**				0.463	0.895
Long protocol	373 (55.3%)	37 (56.1%)	1951 (53.4%)		
Extra long protocol	221 (32.7%)	20 (30.3%)	1203 (32.9%)		
Others	81 (12.0%)	9 (13.6%)	499 (13.7%)		
**Number of transplanted embryos**				< 0.001	0.017
1	31 (4.6%)	2 (3.0%)	472 (12.9%)		
2	644 (95.4%)	64 (97.0%)	3181 (87.1%)		
**Multiple pregnancy (%)**				< 0.001	< 0.001
Yes	483 (71.6%)	47 (71.2%)	792 (21.7%)		
No	192 (28.4%)	19 (28.8%)	2861 (78.3%)		
**Embryo reduction (%)**				0.001	0.004
Yes = 0	17 (2.5%)	3 (4.5%)	35 (1.0%)		
No	658 (97.5%)	63 (95.5%)	3618 (99.0%)		
**Gestational diabetes**				0.034	0.214
Yes	106 (15.7%)	5 (7.6%)	464 (12.7%)		
No	569 (84.3%)	61 (92.4%)	3189 (87.3%)		
**Hypertensive disorder complicating pregnancy**				< 0.001	0.705
Yes	76 (11.3%)	2 (3.0%)	144 (3.9%)		
No	599 (88.7%)	64 (97.0%)	3509 (96.1%)		
**Placenta previa**				< 0.001	< 0.001
Yes	30 (4.4%)	3 (4.5%)	22 (0.6%)		
No	645 (95.6%)	63 (95.5%)	3631 (99.4%)		
**Infant sex**				< 0.001	< 0.001
Male	238 (35.3%)	30 (45.5%)	1749 (47.9%)		
Female	188 (27.9%)	16 (24.2%)	1532 (41.9%)		
Male and female^c^	249 (36.9%)	20 (30.3%)	372 (10.2%)		
**SGA (single birth)**				0.345	–
Yes	9 (5.3%)	0 (0%)	101 (3.8%)		
No	161 (94.7%)	16 (100%)	2528 (96.2%)		
**IUGR (single birth)**				< 0.001	–
Yes	98 (51.6%)	18 (100%)	35 (1.3%)		
No	92 (48.4%)	0 (0%)	2585 (98.7%)		

*SGA* mall for gestational age, *IUGR* intrauterine growth retardation.

^a^Preterm birth group vs. full-term birth.

^b^Early-preterm birth group vs. full-term birth.

^c^In the multiple pregnancy, one is female infant, another is male infant.

Moreover, univariate analysis comparing early preterm birth and full-term birth showed that 10 factors were significantly different between the full-term birth group and the early preterm birth group (*P* < 0.05), including 3 maternal parameters (age, apolipoprotein A1, and thrombin time), 3 pregnancy related factors (multiple pregnancy, embryo reduction, and placenta previa), 3 factors related to the IVF/ICSI procedure (embryo transfer type, treatment cycles and number of embryos transferred), 1 offspring related factor (infant sex) (Tables 1, 2, 3).

### Multivariate analysis

The above 15 detected factors in the univariate analysis were entered into the multivariate logistic analysis. No other factors were considered as confounders. After stepwise regression analysis, multivariate analysis showed that 7 factors (older or younger maternal age, multiple pregnancy, embryo reduction, placenta previa, gestational hypertension, higher triglycerides and shorter activated partial thromboplastin time) were left in the multivariate analysis model for preterm birth. With regard to early preterm birth, the above described 10 factors, see above, were entered into the multivariate analysis. After stepwise regression analysis, 4 factors **(**older or younger maternal age, multiple pregnancy, embryo reduction and placenta previa) remained significant in the multivariate analysis model for early preterm birth (Tables 4, 5).

**Table 4:** Stepwise multivariate logistic regression analysis comparing mothers with preterm and full-term births.

Variables	B	OR	95% CI		*P*
			Lower	Upper	
Maternal age^a^	0.408	1.504	1.108	2.042	0.009
Multiple pregnancy	2.280	9.780	8.014	11.935	< 0.001
Embryo reduction	1.266	3.547	1.736	7.249	0.001
Placenta previa	2.705	14.954	8.053	27.767	< 0.001
Gestational hypertension	0.914	2.494	1.770	3.514	< 0.001
Triglycerides	0.113	1.120	1.011	1.240	0.030
Activated partial thromboplastin time	− 0.034	0.967	0.949	0.985	< 0.001

Fourteen factors showing significant differences in the univariate analysis (see also Tables 1, 2, 3) were entered into the stepwise multivariate logistic regression analysis. Full-term birth group was termed as the reference group of dependent variate. The following seven factors showed no significant effect on preterm birth in the stepwise multivariate logistic regression analysis: Apolipoprotein B, total cholesterol, low density lipoprotein, gestational diabetes, embryo transfer type, number of embryos transferred, and offspring sex.

^a^Maternal age: 40 or 20–24 vs. 25–39.

**Table 5:** Stepwise multivariate logistic regression analysis comparing mothers with early preterm birth and full-term births.

Variables	B	OR	95% CI		*P*
			Lower	Upper	
Maternal age^a^	0.754	2.125	1.049	4.304	0.036
Embryo reduction	1.966	7.145	1.990	25.663	0.003
Multiple pregnancy	2.150	8.588	4.866	15.157	< 0.001
Placenta previa	2.802	16.479	4.381	61.976	< 0.001

Teen factors showing significant differences in the univariate analysis (see also Tables 1, 2, 3) were entered into the stepwise multivariate logistic regression analysis. Full-term birth group was termed as the reference group of dependent variate. The following six factors showed no significant effect on early preterm birth in the stepwise multivariate logistic regression analysis: Apolipoprotein A1, Thrombin time, Treatment cycle, Embryo type, number of embryos transferred, and offspring sex. Reference group was Full-term birth.

^a^Maternal age: 40 or 20–24 vs. 25–39.

The results of the multivariate analyses showed that compared to the maternal age group of 25–39 years, younger mothers (20–24 years) and also older mothers (> 40 years old) displayed a significantly increased preterm birth ratio by 0.50 times (OR = 1.504, 95% CI 1.108–2.042, *P* = 0.009) and early preterm birth ratio by 1.13 times (OR = 2.125, 95% CI 1.049–4.304, *P* = 0.036). Compared to singleton pregnancies, mothers with multiple pregnancies had a significantly increased preterm birth ratio by 8.78 times (OR = 9.780, 95% CI 8.014–11.935, *P* < 0.001) and early preterm birth ratio by 7.58 times (OR = 8.588, 95% CI 4.866–15.157, *P* < 0.001). Embryo reduction significantly increased preterm birth ratio by 2.54 times (OR = 3.547, 95% CI 1.736–7.249, *P* = 0.001) and early preterm birth ratio by 6.14 times (OR = 7.145, 95% CI 1.990–25.663, *P* = 0.003). Placenta previa increased also significantly both preterm birth ratio by 13.95 times (OR = 14.954, 95% CI 8.053–27.767, *P* < 0.001) and early preterm birth ratio by 15.48 times (OR = 16.479, 95% CI 4.381–61.976, *P* < 0.001). The presence of gestational hypertension, higher triglycerides and a shorter activated partial thromboplastin was significantly associated with preterm birth (OR = 2.494, 95% CI 1.770–3.514, *P* < 0.001; OR = 1.120, 95% CI 1.011–1.240, *P* = 0.030; OR = 0.967, 95% CI 0.949–0.985, *P* < 0.001, respectively) but not early preterm birth (*P* > 0.05) (Tables 4, 5).

## Discussion

Our study showed that older (> 39) or younger (< 25) maternal age, multiple pregnancy, placenta previa, and embryo reduction surgery were associated with an increased risk for both preterm birth and early preterm birth after IVF/ICSI. Gestational hypertension, higher triglycerides and a shorter activated partial thromboplastin time were only associated with an increased occurrence of preterm birth but not early preterm birth. However, the lack of some associations in the early preterm group was most likely due to the lower sample size.

The variable “preterm birth” used in this study includes spontaneous and iatrogenic preterm birth. In several studies, it was already shown that the risk of preterm birth among singleton IVF/ICSI pregnancies was significantly higher than that occurring in spontaneous conceptions ^13^. A recent meta-analysis of cohort studies demonstrated that the incidence of preterm birth in IVF/ICSI group and naturally conceived controls are 4.73% and 1.81%, respectively^14^. The underlying risk factors for preterm birth in this population, however, are not fully established so far. The current study, investigating a cohort of women who underwent ART, identified several factors which were associated with preterm birth. The majority of the identified factors, such as maternal age^15^, multiple pregnancies, placenta previa, gestational hypertension, high triglycerides and hypercoagulability^16,17^, have previously been shown to be associated with an increased risk for preterm birth in the general population as well.

Our study showed an increased risk for preterm delivery in association with maternal age. Both younger and older women after ART treatment had an increased risk for preterm birth in our study as it was likewise seen in studies addressing this topic in the general population. Somewhat smaller study done in an ART population mainly reported similar associations ^18^. However, there is also a study with a similar study design as our study showing that women aged 25–29 were at an increased risk for preterm birth in comparison to women aged 30–34. Women aged ≥ 35 years did not display an increased risk of any type of preterm birth^19^. These previous findings may suggest that while there is a positive association between maternal age and the risk for preterm birth, younger women who conceived via ART may display a higher risk for preterm birth compared to older women who conceived undergoing ART. As the proportion of women who conceive with ART also shows an age-related increase, these results may also reflect the increased clinical risk of adverse birth outcomes among young women who needed ART to conceive^18^.

The current study also identified gestational hypertension as risk factor for preterm birth in women who needed ART to conceive. This finding is in line with the current literature, gestational hypertension was shown to be associated with an increased risk for preterm birth in both the general population and women who underwent ART. Moreover, it was shown that ART is associated with a higher frequency of gestational hypertension and preeclampsia as compared to natural pregnancy ^20,21^. The underlying reasons for a higher frequency of gestational hypertension in ART pregnancies remain incompletely understood. Wang et al. showed in a large cohort comparing ART pregnancies to natural conception that ART is associated with a higher prevalence for gestational hypertension, yet this association disappeared once data was stratified by multiple birth cases^22^. The authors concluded that multiple pregnancy which is associated with ART is the single most likely explanation for the increased rate of gestational hypertension among ART mothers.

Another risk factor for preterm birth found by the current study is placenta previa. Placenta previa is associated with preterm birth in the general population as well ^23^. A study that investigated mothers who had conceived both naturally and via ART, showed that the risk of placenta previa was three‐fold higher in the ART pregnancy^24^. The mechanisms underlying this phenomenon still have to be elucidated. It is hypothesized that ART related procedures, such as an induction of uterine contractions due to transcervical catheter insertion or the unique endocrinological environment with high estradiol concentrations following ART cycles might be responsible^25^.

Regarding maternal laboratory parameters recorded before super-ovulation, the current study demonstrated a positive association between maternal triglycerides, shorter activated partial thromboplastin time and preterm birth. Both high triglycerides and hypercoagulability were already shown to be associated with an increased risk for preterm birth in the general population^26,27^. Pregnancy is a hypercoagulable state with an increased thrombotic risk throughout gestation and the postpartum period. Women with thrombophilia may have a further increased risk of placental vascular complications, including pregnancy loss, preeclampsia, intrauterine growth restriction, and placental abruption. Accumulating data suggest that maternal antithrombotic prophylaxis may result in improved gestational outcome^28^. Results from one small prospective study analyzing the relationship between maternal hypercoagulability and preterm labor in 76 women demonstrated a statistically significant procoagulant activity, expressed by a shorter prothrombin time and activated partial thromboplastin time, in pregnant women with premature uterine contractions who gave birth prematurely^27^. The presence of hypercoagulation before starting an IVF treatment was shown to be associated with negative IVF outcomes such as pregnancy loss. However, the mechanism of hypercoagulability associated with preterm birth is not extensively explored. Our study clearly established hypercoagulability as a risk factor for preterm birth in ART and hence may help to clarify ongoing debates on this subject^29^.

Regarding triglycerides, pregnancy is typified by an increase in serum levels of total cholesterol and triglycerides pushed by the rise in estrogen, progesterone and placental lactogen. Existing evidence has demonstrated that high maternal triglycerides levels during pregnancy are related to an increased risk for preterm birth in both obese women and in women with normal BMI in the general population conceiving naturally ^26^. In a retrospective cohort of 2.9 million pregnant women in California, maternal diagnosis of dyslipidemia was significantly associated with increased risk for preterm birth^30^. Also, some studies investigated lipid levels and ART outcomes and showed that maternal triglyceride was inversely associated with live birth rate. However, data regarding the relationship between maternal triglycerides and preterm birth in the setting of ART are scarce^31^. Our findings may motivate further study regarding potential benefits of the treatment of hypercoagulability and hyperlipidemia in pregnancy and its effects on maternal–fetal outcomes.

Our study is in good agreement with previous studies indicating that multiple pregnancy is a strong risk factor for preterm birth, both in the general population as well as in women who conceived trough ART^32^. Multiple pregnancy is considered one of the largest hazards of ART. Until now, ART is associated with a high number of multiple pregnancies due to the current policy transferring multiple embryos simultaneously to achieve a high pregnancy rate^33^. To reduce the risks associated with multiple pregnancy, embryo reduction is performed frequently. Previous meta-analyses have shown that embryo reduction improves outcomes in triplet pregnancies, but never to that degree of singleton pregnancies. An effective method to reduce the risk of multiple births in ART is an elective single embryo transfer, a policy that is adopted by an increasing number of guidelines. However, studies also demonstrated that elective single embryo transfer is not associated with a reduction in the risk for preterm delivery^34^.

Our study has limitations. Firstly, the data of maternal pregestational diabetes, chronic hypertension and PE are missing in the electronic database. We mainly focused on causes or medical reasons for preterm birth. Secondly, only fresh embryo transfer recipients who received IVF/ICSI treatment were included in the current study. It is likely that fresh and frozen embryo transfer present different risks of PTB. We will therefore carry out frozen embryo transfers analyses in the future (“Supplementary information”).

In conclusion, our study demonstrated that maternal age, multiple pregnancy, embryo reduction and placenta previa could increase the risk of preterm birth in women undergoing IVF/ICSI. During ART treatment, the numbers of embryo transfers per cycle should be reduced to two or even to one thus reducing the need for embryo reduction procedures or multiple pregnancy. Strengthening antenatal care is necessary during pregnancy, especially for the patients with placenta previa. The finding that coagulation abnormalities are linked to preterm birth needs independent confirmation and if confirmed may stimulate clinical trials testing drugs interfering with the coagulation system as it is for example done in pregnant women suffering from activated protein C resistance^35^.

## Supplementary Information

Supplementary Information.
