# Supplementary material for: Risk factors associated with preterm birth after IVF/ICSI
Source: Sci Rep. 2022 May 13;12:7944. doi: 10.1038/s41598-022-12149-w (PMC9106684; doi:10.1038/s41598-022-12149-w)
Supplement: Supplementary file 1 — Supplementary Information. [file 41598_2022_12149_MOESM1_ESM.docx]

4340 infertile couples

received IVF/ICSI with live birth

Exclude cases

postterm pregnancy (n=8 )

missing data on gestational age (n=13)

4328 infertile

couples were selected

Full-preterm birth group (n=3653)

Early preterm birth group (n=66)

Preterm birth group

(n=675)

**Supplementary Figure 1.** Study flow chart. Early preterm birth group: gestational age <32 weeks of gestation; preterm birth group: gestational age:<37 weeks of gestation; full-term birth group: ≤ 37 weeks of gestation and <42 weeks of gestation.

**Supplementary Table 1**. Stepwise multivariate logistic regression analysis comparing mothers with early preterm and full-term births

| **Variables** | **B** | **OR** | **95% C.I.** | | ***P*** |
| --- | --- | --- | --- | --- | --- |
|  |  |  | **Lower** | **Upper** |  |
| Maternal age* | 0.754 | 2.125 | 1.049 | 4.304 | 0.036 |
| Multiple pregnancy | 2.150 | 8.588 | 4.866 | 15.157 | <0.001 |
| Embryo reduction | 1.966 | 7.145 | 1.990 | 25.663 | 0.003 |
| Placenta previa | 2.802 | 16.479 | 4.381 | 61.976 | <0.001 |

Ten factors showing significant differences in the univariate analysis (see also tables 1-) were entered into the stepwise multivariate logistic regression analysiscomparing mothers with early preterm and full-term births. The following six factors showed no significant effect on preterm birth in the the stepwise multivariate logistic regression analysis: apolipoprotein A1, thrombin time, blastocyst transfer, treatment cycles and number of embryos transferred, and offspring sex.

*Maternal age: 40 or 20~24 vs. 25-39.
